# C-type natriuretic peptide and natriuretic peptide receptor B signalling inhibits cardiac sympathetic neurotransmission and autonomic function

**DOI:** 10.1093/cvr/cvw184

**Published:** 2016-08-05

**Authors:** Jens Buttgereit, Julia Shanks, Dan Li, Guoliang Hao, Arvinder Athwal, Thomas H. Langenickel, Hannah Wright, Andrey C. da Costa Goncalves, Jan Monti, Ralph Plehm, Elena Popova, Fatimunnisa Qadri, Irina Lapidus, Brent Ryan, Cemil Özcelik, David J. Paterson, Michael Bader, Neil Herring

**Affiliations:** 1 Experimental and Clinical Research Center (ECRC), a joint institution of the Max Delbrück Center for Molecular Medicine (MDC) and the Charité Medical Faculty, Berlin, Germany; 2 Max Delbrück Center for Molecular Medicine (MDC), Campus Berlin-Buch, Robert-Rössle-Strasse 10, 13092 Berlin, Germany; 3 Burdon Sanderson Cardiac Science Centre, Department of Physiology, Anatomy and Genetics, University of Oxford, Parks Road, Oxford OX13PT, UK; 4 Translational Medicine, Clinical Pharmacology and Profiling, Novartis Pharma AG, Basel, Switzerland; 5 Leibniz Institute for Molecular Pharmacology (FMP), Campus Berlin-Buch, Berlin, Germany; 6 Helios Clinic Bad Saarow, Pieskower Strasse 33, Bad Saarow, Germany

**Keywords:** Natriuretic peptides, Sympathetic, Norepinephrine, Calcium, Hypertension

## Abstract

**Aims:**

B-type natriuretic peptide (BNP)–natriuretic peptide receptor A (NPR-A) receptor signalling inhibits cardiac sympathetic neurotransmission, although C-type natriuretic peptide (CNP) is the predominant neuropeptide of the nervous system with expression in the heart and vasculature. We hypothesized that CNP acts similarly to BNP, and that transgenic rats (TGRs) with neuron-specific overexpression of a dominant negative NPR-B receptor would develop heightened sympathetic drive.

**Methods and results:**

Mean arterial pressure and heart rate (HR) were significantly (*P* < 0.05) elevated in freely moving TGRs (*n* = 9) compared with Sprague Dawley (SD) controls (*n* = 10). TGR had impaired left ventricular systolic function and spectral analysis of HR variability suggested a shift towards sympathoexcitation. Immunohistochemistry demonstrated co-staining of NPR-B with tyrosine hydroxylase in stellate ganglia neurons. In SD rats, CNP (250 nM, *n* = 8) significantly reduced the tachycardia during right stellate ganglion stimulation (1–7 Hz) *in vitro* whereas the response to bath-applied norepinephrine (NE, 1 μM, *n* = 6) remained intact. CNP (250 nM, *n* = 8) significantly reduced the release of ^3^H-NE in isolated atria and this was prevented by the NPR-B antagonist P19 (250 nM, *n* = 6). The neuronal Ca^2+ ^current (*n* = 6) and intracellular Ca^2+ ^transient (*n* = 9, using fura-2AM) were also reduced by CNP in isolated stellate neurons. Treatment of the TGR (*n* = 9) with the sympatholytic clonidine (125 µg/kg per day) significantly reduced mean arterial pressure and HR to levels observed in the SD (*n* = 9).

**Conclusion:**

C-type natriuretic peptide reduces cardiac sympathetic neurotransmission via a reduction in neuronal calcium signalling and NE release through the NPR-B receptor. Situations impairing CNP–NPR-B signalling lead to hypertension, tachycardia, and impaired left ventricular systolic function secondary to sympatho-excitation.

## 1. Introduction

Natriuretic peptides (NPs) comprise a family of structurally related, but genetically distinct peptide hormones, including atrial NP (ANP), brain/B-type NP (BNP), and C-type NP (CNP).^1^ ANP and BNP levels are greatly elevated in heart failure,^2^ and are also negative prognostic indicators during hypertension,^3^ and acute ischemic stroke.^4^ They are both predominantly synthesized in the cardiac chambers, and released upon cardiac wall stretch into the circulation, modulating vascular tone, permeability, renal function, and the renin–angiotensin system.^1^^,^^5^ Locally within the heart, ANP and BNP can also act directly on cardiomyocytes, protecting against cardiac hypertrophy and heart failure.^3–7^ More recently, BNP has been shown to inhibit local cardiac sympathetic neurotransmission by reducing calcium signalling in post-ganglionic sympathetic neurons which may be beneficial during chronic heart failure.^6^

C-type NP plasma concentrations are comparatively low compared with ANP/BNP, but they also increase during heart failure,^8^^,^^9^ and therefore CNP may also play an important role in the pathophysiology of this condition. CNP was originally identified in porcine brain and is considered the predominant NP in the nervous system,^10–12^ although it is also expressed locally within the heart and coronary vasculature.^13^ However, it is not known whether CNP, like BNP, is able to modulate cardiac sympathetic neurotransmission. The NPs mediate their actions through cell surface receptors.^14^ ANP and BNP bind specifically to natriuretic peptide receptor A (NPR-A), whereas CNP specifically activates NPR-B. Both receptors have intrinsic guanylyl cyclase activity, resulting in an increase in cGMP^15^ and protein kinase G signalling.^16–18^ Although the cardiac phenotypes of NPR-A signalling have been well studied within transgenic and disease models, little is known about the cardiac signalling of NPR-B. NPR-B homozygous knockout mice display seizures, female sterility, and priapism, making them un-suitable for detailed cardiovascular phenotyping.^19^^,^^20^ To overcome this limitation, we generated a transgenic rat (TGR) with neuron-specific overexpression of a dominant-negative NPR-B mutant (NPR-BΔKC) under the control of a promoter for neuron-specific enolase (NSE-NPR-BΔKC).^18^ We hypothesized that this TGR would display elevated sympathetic nerve activity compared with its SD control *in vivo*, and investigated the cellular mechanisms by which CNP influences cardiac sympathetic neurotransmission in the SD rat *in vitro*.

## 2. Methods

A detailed methods section can be found in the Supplementary material online. Experiments conformed to the Guide for the Care and Use of Laboratory Animals published by the US National Institutes of Health (NIH Publication No. 85-23, revised 2011) and the Animals (Scientific Procedures) Act 1986 (UK). Experiments were performed under British Home Office Project License PPL 30/2360 and the German License LaGeSo G0343/08. For all *in vitro* experiments, rats were euthanized by approved home office schedule 1 method of overdose by pentobarbital (0.3 mL, 100 g) under deep anaesthesia (3% isoflurane and 97% oxygen).

### 2.1 Statistical analysis

Data are presented as mean ± S.E.M. (standard error of the mean). Differences between two groups were evaluated by using an unpaired Student’s *t*-test. Where multiple interventions are made in each of the two groups, a two-way analysis of variance followed by Sidak’s multiple comparison test was performed using PRISM^®^ software (PRISM, GraphPad Software Inc, San Diego, California, USA). The significance level was accepted at *P *< 0.05.

## 3. Results

### 3.1 Hypertension, tachycardia, and evidence for autonomic dysfunction in the TGR

**Figure 1:**
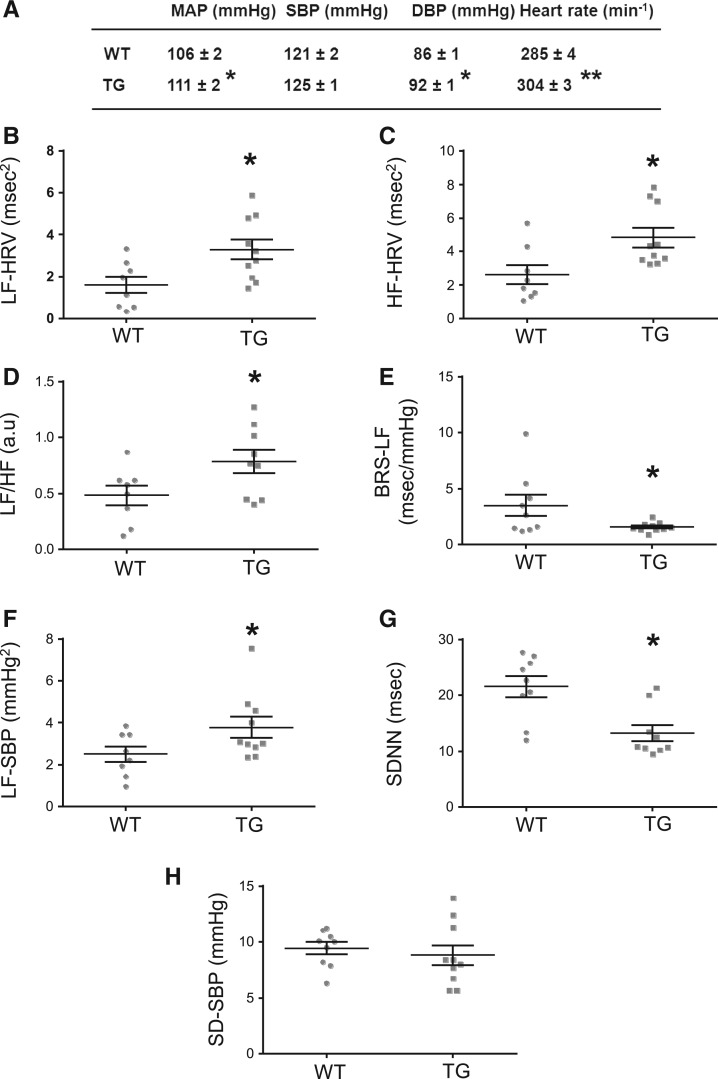
Telemetric measurements and powerspectral analysis in conscious SD wildtype (WT, *n* = 9) and pNSE-NPR-BΔKC transgenic (TG, *n* = 10) rats. (*A*) MAP, SBP, and diastolic blood pressure (DBP) as well as HR in SD and TG rats. (*B*–*D*) Cumulative spectral moduli of HRV and SBP in the LF and HF bands in control and NSE-NPR-BΔKC TG rats. (*E*, *F*) Spontaneous BRS-LF and calculated as the gain of the LF-SBP. (*G*, *H*) Overall HRV was assessed as the standard deviation of the NN interval (SDNN) along with the standard deviation of systolic blood pressure (SD-SBP) (*P < 0.05, **P < 0.01 transgene vs. SD wild-type).

Telemetric blood pressure measurement in 4-month-old conscious rats revealed an elevated mean (MAP) and diastolic blood pressure in TGR (*n* = 10) compared with SD controls (*n* = 9). Furthermore, heart rate (HR) was significantly elevated in the TGR group (*Figure**1**A*).

Transthoracic echocardiography of TGR (*n* = 9) revealed significantly reduced left ventricular systolic function (LVSF) indicated by a reduced ejection fraction, reduced fractional shortening, and increased left ventricular end-systolic diameter, whereas left ventricular end-diastolic dimensions were similar to those of SD (*n* = 9) wild-type animals (*Table 1*).

**Table 1:** Transthoratic echocardiography in TGR (TG; *n* = 9) and SD wild type (WT; *n* = 9)

	LVEDD (mm)	LVESD (mm)	FS (%)	EF (%)
WT	8.3 ± 0.2	4.8 ± 0.2	42 ± 2	72 ± 2
TG	8.2 ± 0.2	5.6 ± 0.1**	32 ± 1***	58 ± 2***

LVEDD, left ventricular end-diastolic dimension; LVESD, left ventricular end-systolic dimension; FS, fractional shortening; EF, ejection fraction.

** *P* < 0.01,

*** *P* < 0.001.

To investigate the influence of neural NPR-B signalling on cardiovascular autonomic control, spectral analysis of blood pressure and heart rate variability (HRV) was performed. Overall HRV as measured by the standard deviation of the NN interval was reduced in the TGR compared with the wild-type although the standard deviation of systolic blood pressure (SBP) variability was similar (*Figure* *1**G and H*). The low frequency (LF) and high frequency (HF) peaks of HRV and the LF peak of SBP as well as the LF/HF ratio were higher in the TGR compared with SD controls. Cross-spectral analysis revealed decreased baroreflex sensitivity in the low frequency (BRS-LF) band in the TGR compared with SD controls (*Figure* *1**B–F*). This suggests an imbalance of the autonomic nervous system with a shift to higher levels of sympathetic drive relative to parasympathetic activity. In keeping with this observation, plasma renin activity and concentration as well as plasma angiotensin II concentrations were also significantly increased in the TGR compared with SD controls (*Table 2*).

**Table 2:** Plasma renin activity (PRA) and concentration (PRC) and plasma angiotensin II concentration (ATII) in TGR (TG, *n* = 9) and SD wild type (WT, *n* = 10)

	PRA (µg/mL × h)	PRC (ng/mL)	ATII (pg/mL)
WT	8.1 ± 1.9	11.8 ± 2.4	104 ± 14
TG	15.8 ± 2.4*	24.6 ± 3.5**	156 ± 10*

* *P* < 0.05,

** *P* < 0.01.

### 3.2 CNP reduces the HR response to sympathetic stimulation by inhibiting norepinephrine release

**Figure 2:**
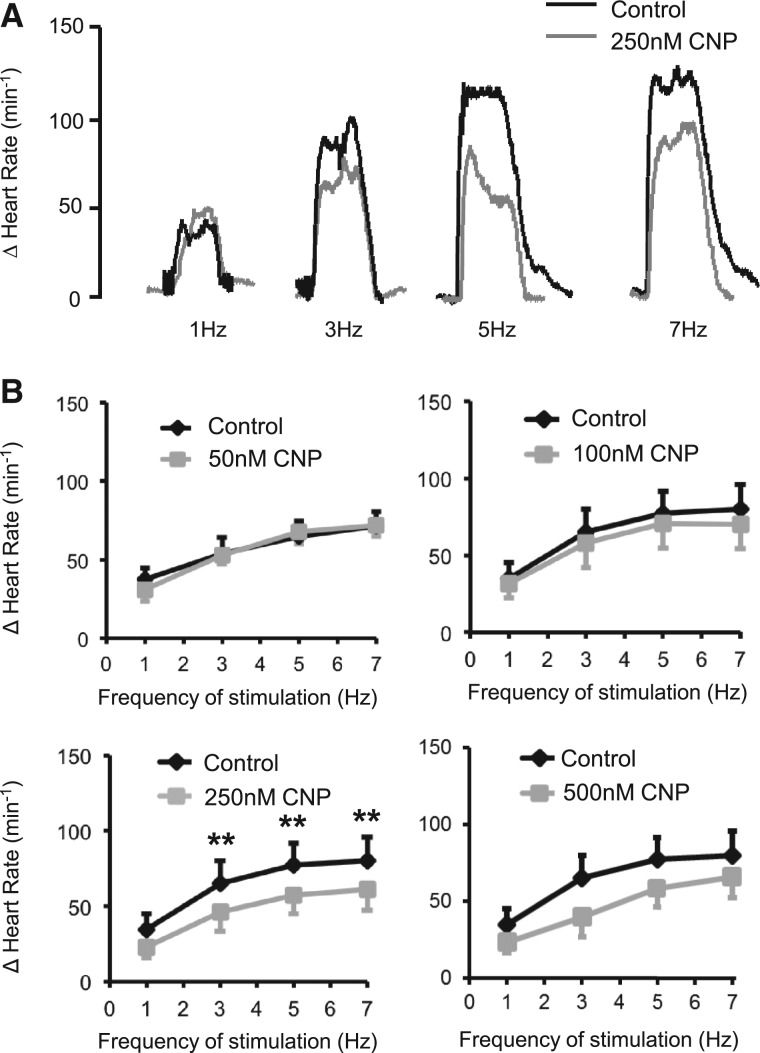
(*A*) Representative raw data traces showing the effect of 250 nM CNP (grey) compared with control (black) on the HR response to stimulation of the right stellate ganglion at 1–7 Hz in isolated atria-stellate preparations from the SD rat *in vitro*. (*B*) Group mean data showing the effect of CNP at 50 (*n* = 7), 100 (*n* = 8), 250 (*n* = 8), and 500 (*n* = 6) on the tachycardia to right stellate stimulation at 1–7 Hz (**P < 0.01 control vs. CNP).

**Figure 3:**
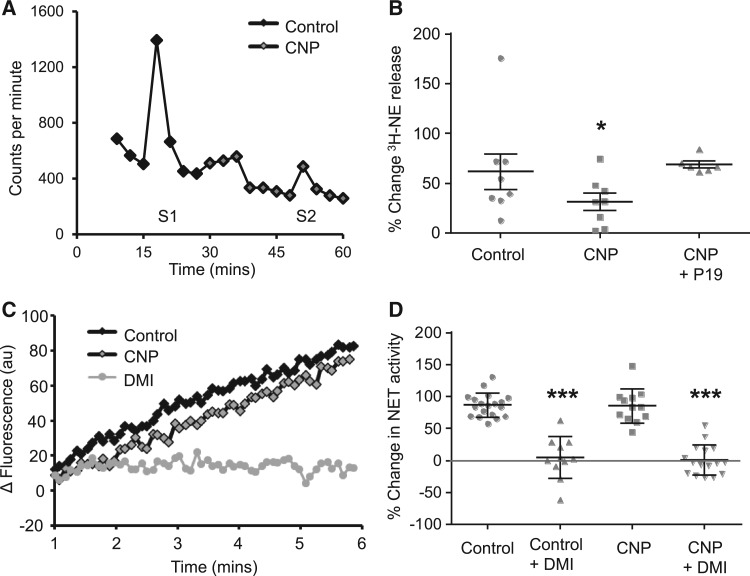
(*A*) Representative raw data trace showing ^3^H efflux in counts per minute over time and the effect of 250 nM CNP on the evoked release of ^3^H-NE (S2) in response to field stimulation (5 Hz) compared with control (S1) in right atria from the SD rat. (*B*) Group data (including mean and standard error) as a dot plot showing a significantly reduced release of ^3^H-NE (percentage increase from baseline) in response to field stimulation following addition of 250 nM CNP (*n* = 8 atria). The action of CNP is lost in the presence of the NPR-B antagonist P19 (250 nM, *n* = 6 atria). (*C*) Raw data trace showing increase in fluorescence (au) over time, of a stellate sympathetic neuron in a solution containing the NE reuptake transporter (NET) assay. The control slope is represented in black and the slope after addition of 250 nM CNP in black with grey fill. The fluorescence increase is blocked by the NET inhibitor desipramine (DMI, 1 μM, in grey). (*D*) Group data (including mean and standard error) as a dot plot showing no change in NET activity between CNP 250 nM and control. DMI blocks fluorescence uptake in both groups. (Control, *n* = 19 neurons; CNP *n* = 12 neurons). *P < 0.05 control vs. CNP, ***P < 0.001 vs. control or CNP.

To evaluate whether CNP could modulate cardiac sympathetic neurotransmission, we used isolated atria right stellate ganglion preparations from SD rats. After equilibration the beating rate was 263 ± 7 bpm (*n* = 29) and CNP (50–250 nM) did not significantly change this baseline. The HR response to stimulation of the right stellate ganglion was unchanged by 50 (*n* = 7) or 100 nM CNP (*n* = 8) but was significantly reduced by CNP at 250 nM (*n* = 8), with a trend to reduce the response at 500 nM (*n* = 6) (*Figure**2**A and B*). However, CNP did not affect the HR response to exogenous norepinephrine (NE 1 µM, control +125 ± 20 bpm, 500 nM CNP +115 ± 15 bpm, *n* = 6). This suggests that CNP reduces the HR response to sympathetic stimulation via a neural mechanism. We therefore directly measured NE release and reuptake to see whether CNP modulates these processes. CNP (250 nM) significantly reduced the release of ^3^H labelled NE to field stimulation in isolated right atria from the SD rat (*n* = 8) (*Figure**3**A and B*), although this could be prevented by the NPR-B antagonist P19 (250 nM, *n* = 6). CNP had no effect on the function of the NE reuptake transporter when measured in isolated sympathetic stellate ganglia neurons (control, *n* = 19; CNP 250 nM, *n* = 12) (*Figure**3**C and D*). The NE content of unstimulated stellate ganglia was also unchanged by 250 nM CNP (control, *n* = 6, 1.25 ± 0.05 vs. CNP, *n* = 6, 1.36 ± 0.66 pmol/mg tissue) suggesting that CNP did not modulate NE synthesis. In summary, these data provide evidence for a sympathoinhibitory action of CNP acting on post-ganglionic neurons from the right stellate ganglion to reduce sympathetic neurotransmission.

### 3.3 Endogenous NPR-B expression and modulation of neuronal calcium handling by CNP in stellate sympathetic neurons

**Figure 4:**
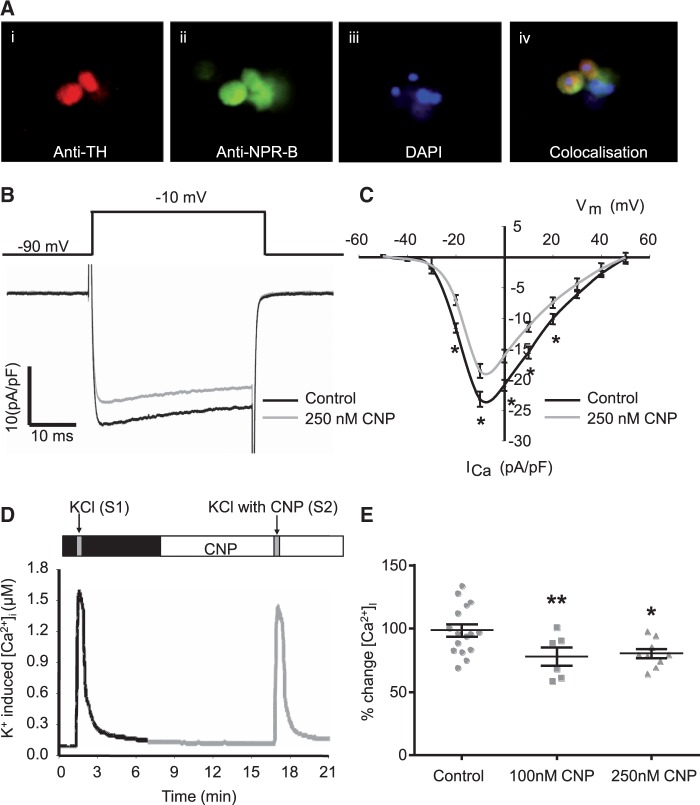
(*A*) Immunohistochemistry showing tyrosine hydroxylase (TH) staining with texas red in cultured sympathetic neurons of the right stellate ganglia from SD rats also staining positive for the NPR-B in green (fluorescein) Nuclear staining is shown with DAPI in blue and co-localization is demonstrated by overlap of staining in yellow (×20 magnification). (*B*) Representative whole cell calcium current density traces with (grey S2) or without (black) 250 nM CNP. Current evoked by test pulses from a holding potential of − 90 to − 10 mV. (*C*) Mean current density–voltage relations in the presence or absence of 250 nM CNP (*n* = 6). (*P < 0.05, **P < 0.01 control vs. CNP). (*D*) Representative raw data trace of calcium transients induced by 50 mM KCl (30 s) in a single stellate sympathetic neuron in control (black S1) and in the presence of 100 or 250 nM CNP (grey). (*E*) Group data (including mean and standard error) as a dot plot showing a significant reduction in KCl-evoked calcium transients in isolated stellate neurons from the SD rat after 100 nM (*n* = 6) and 250 nM (*n* = 9) CNP. *P < 0.05, **P < 0.01 vs. control.

Immunohistochemistry revealed that endogenous NPR-B is colocalized to tyrosine hydroxylase containing sympathetic neurons cultured from the stellate ganglia of SD rats (*Figure**4**A*). Within isolated stellate sympathetic neurons CNP (250 nM, *n* = 6) significantly reduced the magnitude of the neuronal calcium current measured by patch clamp recording (*Figure**4**B and C*). CNP (100 nM, *n* = 6 and 250 nM, *n* = 9) also significantly reduced the magnitude of depolarization-induced intracellular calcium transients (*Figure**4**D and E*).

### 3.4 Effect of reducing sympathetic neurotransmission in the TGR

**Figure 5:**
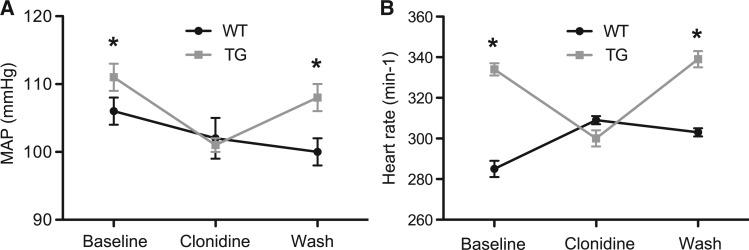
Telemetric measurement in freely moving conscious NSE-NPR-B&Delta;KC TGR (TG) and SD wild type (WT) following clonidine treatment. Clonidine significantly reduced MAP (*A*), and HR (*B*). After a washout phase, parameters were indistinguishable from baseline values (*n* = 9 per group. *P < 0.05 vs. baseline).

To test whether increased sympathetic activity is responsible for the hypertension and tachycardia observed in the pNSE-NPR-BΔKC transgenic animal, the TGR were treated with the sympatholytic drug clonidine (125 µg/kg × day). Clonidine treatment significantly reduced MAP and HR to the level detected in SD wild-type animals. After washout of clonidine, haemodynamic parameters were indistinguishable from baseline values (*Figure**5**A and B*), indicating increased sympathetic activity in NSE-NPR-BΔKC TGR (*n* = 9, both groups).

## 4. Discussion

Dysregulation of the autonomic nervous system has previously been implicated in human^21–24^ and animal models of cardiovascular disease and heart failure.^25–28^ We therefore investigated the impact of reduced neuronal NPR-B signalling on hemodynamic parameters. We generated a novel rat model with neuron-specific overexpression of a dominant-negative mutant of NPR-B.^18^ The model allowed us to overcome the limitations related to knockout of the NPR-B gene and demonstrated for the first time that NPR-B and its ligand CNP are involved in the regulation of cardiovascular function via direct sympathoinhibition. We studied the function of neuronal NPR-B on the autonomic regulation of the cardiovascular system *in vivo* in the TGR, and the *in vitro* effect of CNP on SD wild-type tissue. Our novel findings were that CNP signalling via the NPR-B receptor is sympathoinhibitory via a direct action on stellate sympathetic neurons. CNP reduces the neuronal calcium current and intracellular calcium transient to inhibit NE release, although CNP does not alter NE re-uptake via the NET1 transporter, or NE synthesis.

Telemetric measurement in conscious rats revealed increased mean arterial blood pressure, HR, and changes in HRV consistent with increased sympathetic relative to parasympathetic activity in the transgenic model. Conversely, the HR response to stimulation of the right stellate ganglion *in vitro* was significantly reduced by CNP in wild-type SD rats, although CNP did not alter the HR response to NE, suggesting that CNP acts pre-synaptically to inhibit sympathetic neurotransmission rather than post-junctionally on cardiomyocyte β_1_ receptor signalling. This was confirmed by a reduced release of tritiated NE upon field stimulation in the isolated right atria by CNP, an effect that could be prevented by an NPR-B antagonist. These findings mirror observations made with BNP and NPR-A signalling which can also reduce postganglionic sympathetic release of NE in the heart.^6^

Immunohistochemical studies confirmed the presence of NPR-B on stellate sympathetic neurons of the SD rat. The reduced tritiated NE release is coupled with no change in the rate of action of the NE reuptake transporter; therefore, increased NET activity is not responsible for the reduced neurotransmitter release at the cellular level in response to NPR-B activation. Moreover, within isolated stellate sympathetic neurons we have shown that CNP reduces neuronal calcium current and depolarization-induced intracellular calcium transients. NPR-B is proposed to act via particulate guanylyl cyclase, resulting in an increase in cGMP^15^ and protein kinase G signalling,^16–18^ leading to the inhibition I_ca._^29^ This pathway has previously been studied in relation to NPR-A/BNP signalling in stellate sympathetic neurons where cGMP–protein kinase G-dependent inhibition of calcium handling and neurotransmission is limited by PDE2A.^6^ Our results demonstrate a direct effect for CNP signalling on postganglionic stellate sympathetic neurons to reduce neurotransmission. Others have demonstrated that CNP can increase tyrosine hydroxylase mRNA expression and catecholamine content via a cGMP-dependent pathway in bovine adrenal chromaffin cells.^30^ We therefore also measured the action of CNP on NE synthesis, although we found no effect on NE content of unstimulated stellate ganglia.

Previously, a modulatory effect of CNP on sympathetic cotransmission in the rat isolated tail artery has also been shown,^31^ and we cannot exclude the possibility that vasoconstriction may contribute to the hypertensive phenotype of the neural NPRB-TGR due to increased vascular sympathetic neurotransmission and/or increased activation of the renin–angiotensin system by the sympathetic nervous system.

Within the TGR the neural action of NPR-B signalling was confirmed by the administration of the sympathetic blocker clonidine.^32^ Clonidine supresses sympathetic outflow at least in part by stimulating central pre-synaptic α_2_ receptors to reduce NE release.^32^ Clonidine reduced mean arterial blood pressure and HR of the transgenic model back to that of wildtype, before returning to the baseline of the TGR after a washout period. This reinforces the principle that the hypertension and tachycardia present in the TGR are due to an increased sympathetic drive. This hypothesis is further supported by observations showing that NPR-B is the predominant NPR in the heart, and is down-regulated in heart failure.^33^^,^^34^ Furthermore, previous studies have shown that cardiac cells are actively involved in the production of CNP and cardiomyocyte-specific overexpression in a transgenic mouse model prevented cardiac hypertrophy induced by myocardial infarction.^35^^,^^36^ It is interesting to note that a ubiquitous overexpression of the dominant negative NPR-B mutant leads to concentric left ventricular hypertrophy and increased sympathetic activity,^18^ whereas we report here that neural-specific over expression of the mutant leads to impaired LVSF without hypertrophy. Cardiac myocyte NPR-B signalling may therefore inhibit cardiac hypertrophy as has been demonstrated for NPR-A,^37^ but this might be deleterious in the presence of sympathetic hyperactivity which may then promote hypertensive heart failure. The impaired LVSF observed in the TGR may cause further sympatho-excitation through activation of arterial baroreceptors as heart failure progresses. If the sympathoexcitation in the TGR was purely secondary to systolic heart failure, it would be expected that the TGR would have normal or low blood pressure. The fact that blood pressure is paradoxically *elevated* in the TGR points to sympathoexcitation being the main driver of the systolic heart failure rather than the other way around.

We also observe increased plasma renin levels and activity, and higher circulating levels of angiotensin II in the TGR which can be both caused by renal sympathetic stimulation and by renal hypoperfusion during heart failure. Angiotensin II promotes the release of NE from cardiac sympathetic nerve terminals,^27^ so this may also act as a positive feedback mechanism increasing sympathetic drive and worsening subsequent heart failure. We think it highly unlikely that the transgene itself is acting directly on cardiac myocytes to induce a cardiomyopathy as we have previously demonstrated by reverse transcriptase-polymerase chain reaction that the transgene is present in neuronal tissue only and not heart, lung, aorta, or kidney.^38^ We have observed impaired LVSF and abnormal HRV in the TGR compared with the SD control at 8 weeks of age, but it is not known whether the abnormal HRV precedes the left ventricular dysfunction in the immediate postnatal period.

## 5. Limitations

Although our studies have focused on the role of CNP in modulating the peripheral sympathetic nervous system at the level of the heart, CNP is the major NP in the central nervous system and cerebrospinal fluid.^12^^,^^39^ NPR-B expression has been observed in a broad range of brain regions, including brainstem nuclei involved in autonomic function.^40^^,^^41^ We therefore cannot exclude a central action of CNP in the NPR-B TGR whereby CNP acts to inhibit pre-ganglionic sympathetic outflow. NPR-B has also been reported in glial cells in the central nervous system^42^ although we only observe NPR-B staining in TH positive neurons in the stellate which are morphologically distinct from glial cells or fibroblasts. It is also possible that CNP may facilitate vagal neurotransmission. In the guinea pig BNP and also CNP can facilitate vagal neurotransmission, thereby inducing bradycardia via a cGMP-dependent mechanism, comparable to NO.^43^ However, in SD rats we find no NPR-B staining in cholinergic neurons in the right atria (data not shown).

Heart rate variability is a controversial method of providing a quantitative measure of the contribution of the individual branches of the autonomic nervous system, particularly when the LF/HF ratio is viewed in isolation (e.g. see the point–counterpoint debate in the *Journal of Applied Physiology*^44^). However, we report HRV data for LF and HF bands individually as well as a ratio, and also present data on spontaneous BRS-LF and calculated as the gain of the transfer function between systolic blood pressure (SBP) and pulse interval (LF-SBP). We cautiously conclude that all of these indices, together with the changes in mean arterial pressure (MAP) and HR, are evidence for a change in autonomic balance and are careful not to attribute this solely to a particular branch of the autonomic nervous system based on these data alone. We perform further experiments on isolated hearts and stellate neurons, measure plasma renin/angiotensin levels, and use clonidine treatment *in vivo* to extend these findings.

The ^3^H method of quantifying NE release also has limitations. We deliberately chose the same stimulation parameters used in the isolated atrial/stellate preparation which produced physiological increases in HR (5 Hz, 20 V, 1 ms pulse duration), although this will not exactly mimic the *in vivo* situation. The measured release of NE has been shown to be frequency dependent and also dependent on extracellular calcium and blocked by N-type calcium channel blockers.^45^

Although we clearly show the action of exogenous CNP on cardiac neurotransmission in SD controls, we have not demonstrated an impaired action of CNP on neuronal calcium signalling, NE release, and re-uptake in the TGR. Although an NPR-B antagonist prevents that action of CNP on NE release in the SD control, it is possible that compensatory upregulation of the NPR-A receptor or the clearance receptor NPR-C influences the phenotype of the TGR. We have however previously shown that the NPR-B TGR has impaired generation of cGMP in neuronal tissue in response to CNP, whereas cGMP generation via NPR-A remains intact.^38^ In addition to this, inhibiting sympathetic drive with clonidine normalizes the difference in blood pressure and HR between the NPR-B TGR and the SD providing some evidence for its role in producing these differences.

The levels of circulating CNP are also far lower than the 100 nM (and above) used in our *in vitro* experiments. However, the circulatory levels of most hormones are orders of magnitude lower than local concentrations at neural release sites and the concentrations of exogenous ligand that produce responses in isolated tissues. For example, circulatory NE is in the low nanomolar range,^46^ whereas exogenous concentrations required for maximal stimulation of isolated cardiac tissue are in the micromolar range.^27^

In summary, our study provides the first evidence that impaired neuronal NPR-B signalling leads to sympathoexcitation, hypertension, tachycardia, and impaired LVSF. The findings suggest that activation of NPR-B or its downstream signalling pathways might be beneficial in the treatment of cardiovascular diseases associated with autonomic dysfunction. We believe that these observations could lead to further therapeutic avenues.

## Supplementary material

Supplementary material is available at *Cardiovascular Research* online.

Supplementary Data
