# Supplementary material for: C-type natriuretic peptide and natriuretic peptide receptor B signalling inhibits cardiac sympathetic neurotransmission and autonomic function
Source: Cardiovasc Res. 2016 Aug 5;112(3):637–44. doi: 10.1093/cvr/cvw184 (PMC5157132; doi:10.1093/cvr/cvw184)
Supplement: Supplementary Data [file supp_cvw184_cvw184.DC1.html]

Supplementary Data | Cardiovascular Research

## Supplementary Data

files

- Supplementary Data - docx file
