# Supplementary material for: C-type natriuretic peptide and natriuretic peptide receptor B signalling inhibits cardiac sympathetic neurotransmission and autonomic function
Source: Cardiovasc Res. 2016 Aug 5;112(3):637–44. doi: 10.1093/cvr/cvw184 (PMC5157132; doi:10.1093/cvr/cvw184)
Supplement: Supplementary Data [file CNP_paper_R3_online_methods.docx]

**Online methods supplement**

***Generation of transgenic rats***

Sprague Dawley (SD) rats with ubiquitous overexpression of the dominant negative NPR-BΔKC mutant were generated as previously described [^1^](#_ENREF_1). For generating SD rats (Taconic, Denmark) with neuron-specific overexpression of NPR-BΔKC, cDNA encoding the dominant-negative mutant with C-terminal Flag epitope was cloned under the control of a promoter for neuron-specific enolase (the vector was a generous gift from K.H. Braunewell, Ruhr-Universität-Bochum, Germany). pNSE-NPR-BΔKC-Flag was linearized and microinjected into the male pronucleus of rat zygotes as previously described [^2^](#_ENREF_2). Founder animals were identified by southern blot, using ^32^P-labeled NPR-BΔKC and bred to homozygosity. Neurons of these transgenic rats (TGR) express functional dimers of the NPR-B receptor containing the dominant-negative NPR- BΔKC mutant and show impaired CNP stimulated cGMP production whist ANP stimulated cGMP production remains intact [^3^](#_ENREF_3).

***Haemodynamic evaluation and spectral analysis of arterial blood pressure and heart rate.***

For telemetric blood pressure monitoring in conscious rats, SD (n=9) and TGR (n=10) at 16 weeks of age were anesthetized with ketamine (10mg/100g body weight) and xylazine (0.02mg/100g body weight). A pressure transducer (DSI, USA) was implanted into the abdominal cavity and the catheter was anchored in the abdominal aorta and rats allowed to recover for 15 days before data recording. After recovery, data were recorded under basline conditions, followed by clonidine treatment (125 µg/kg x day) in the drinking water, and a washout phase. Spectral analysis of blood pressure and heart rate variability was performed as described elsewhere [^4^](#_ENREF_4). The baroreceptor heart rate reflex and HRV in both low and high frequency ranges (LF-HRV and HF-HRV) were investigated using spontaneous changes in BP and HR in freely moving rats. The power spectra of SBP, pulse interval time series, and the cross spectra were calculated using fast Fourier transformation (FFT). Low-frequency components of pulse intervals spectrum (LF), total power and the baroreflex gain (BRS-LF) were calculated. Data segments ~100 seconds were used for spectral analysis. Linear trends were removed and power spectral density was estimated with the FFT-based Welch algorithm using segments of 512 data points with 50% overlapping and Hanning window. The power in the frequency ranges of low frequencies (LF: 0.25 to 0.6 Hz) and high frequencies (HF: 1.0 to 3.0 Hz) was calculated. Five representative intervals were chosen for spectral analysis and averaged according to the following criteria 1) steady state conditions, 2) no large sudden BP changes, 3) no artifacts. The frequency bands were adapted for analysis in rats considering the ranges of HR and breathing frequencies [^4^](#_ENREF_4)^,^ [^5^](#_ENREF_5). Overall heart rate variability was assessed as the standard deviation of the NN interval (SDNN) and blood pressure variability as the standard deviation of SBP (SD-SBP).

***Transthoracic echocardiography***

Transthoracic two dimensional M-mode echocardiography was performed in transgenic and SD rats at 16 weeks of age under anesthesia with isoflurane, by using an Acuson Sequoia C256 echocardiograph with a 14-MHz probe (Siemens) as previously described [^1^](#_ENREF_1).

***Measurement of plasma renin concentration, plasma renin activity and plasma angiotensin II levels***

Venous blood was obtained under anaesthesia and transferred to chilled tubes containing 5% phenanthroline-EDTA. Plasma was collected after centrifugation at 10,000g at 4°C for 10minutes and placed on dry ice. Plasma renin activity (PRA), -concentration (PRC) and angiotensin II concentrations were determined using radioimmunoassay as previously described [^6^](#_ENREF_6).

***Isolated sinoatrial node/right stellate ganglion preparation***

The spontaneously beating atria with intact sympathetic innervation were isolated from 16 week old SD rats and transferred to a preheated (37±0.2 °C), water-jacketed organ bath containing 60 ml Tyrode solution, containing (mM) NaCl 120, KCl 4.7, MgSO_4_ 1.2, NaHCO_3_ 25, CaCl_2_ 2, KH_2_PO_4_ 1.2 and glucose 11, and was aerated with 95 % O_2_/5 % CO_2_ (pH 7.4). The method for dissecting and measuring responses to sympathetic nerve stimulation has been described previously [^7^](#_ENREF_7). Before starting each protocol, the mounted atria were allowed to equilibrate for 45-60 minutes until beating rate stabilized (±5 beats per minutes, bpm, over 20 minutes). The stellate was stimulated at 1, 3, 5 and 7Hz, (20V, 1 ms pulse duration for 30 s). Drugs were applied directly to the organ bath and incubated until a consistent response to sympathetic nerve stimulation (SNS) was obtained.

***^3^H-Norepinephrine release to field stimulation from right atrial preparations***

The spontaneously beating right atrium from 16 week old SD rats was isolated and transferred to a preheated (37±0.2 °C), continuously oxygenated, water-jacketed organ bath containing 3 ml Tyrode solution where the atrium was pinned flat over a silver stimulating electrode. The method for determining the local release of ^3^H-NE to field stimulation 5 Hz (20 V, 1 ms pulse width, for 1 minutes) has been previously described [^7^](#_ENREF_7). Briefly, following a 20-min equilibration period, the double atria preparation was incubated with 5 μM ^3^H-NE (0.185 MBq, ARC) and ascorbic acid (30 μM; Sigma). The atria were field stimulated at 5Hz (20V, 1ms pulse duration) for 10 s every 30 s, for 30 min to facilitate uptake of ^3^H-NE into the transmitter stores of the presynaptic terminal. Following ^3^H-NE incubation, excess radioactivity was washed from the preparation with Tyrode solution superfusion for 45 min at a rate of 3 ml/min. Bath solution was then replaced every 3 min for 60 min. A 0.5-ml sample from each solution change was added to 4.5 ml scintillation liquid (Ecoscint A; National Diagnostics), and the amount of radioactivity was measured [counts/min (CPM)] using a liquid scintillation counter (Tri-Carb 2800TR, Perkin-Elmers life science). At 16 min the atria were stimulated at 5 Hz for 1 min [stimulation 1 (S1)]. The bath solution from 27 min onwards was changed to contain CNP. A second stimulation (S2) was applied at 49 min. The ^3^H-NE outflow was calculated as a proportional percentage increase: ^3^H-NE outflow = {[CPM(y) - CPM(x)]/CPM(x)} x100, where CPM(x) is CPM immediately before stimulation and CPM(y) is CPM immediately after stimulation. Previous control experiments have demonstrated no significant change in the magnitude of the response over two stimulations [^8^](#_ENREF_8).

***Norepinephrine content of stellate ganglia***

Both right and left stellate ganglia were dissected from SD rats at 4 weeks of age and incubated in control media or media containing CNP (250nM) for 15minutes before being snap frozen in liquid nitrogen. On the day of analysis, stellate pairs from an individual animal were weighed and then homogenised in 250μl of perchloric acid (0.1M) and centrifuged through a 0.22-mm filter (VWR) at 13,000g for 3min at 4°C. Samples (100μl) were injected onto an isocratic HPLC system and quantified using an LC-4B electrochemical detector and a carbon working electrode held at +0.7V vs a Ag/AgCl reference electrode (Decade SDC, Antec). HPLC separation was performed using a 250-mm Microsorb C18 reverse-phase column (Agilent) and a mobile phase comprising methanol (13%v/v), NaH2PO4 (120mM), EDTA (0.8mM), and sodium octane sulfonate (3.2 mM), pH 3.27, and the flow rate was fixed at 1ml/min. Catecholamine content was determined relative to authentic, freshly prepared standards and normalized to sample weight, as previously described [^9^](#_ENREF_9).

***Primary Cultures of Dissociated Sympathetic Neurons.***

Sympathetic neurons were isolated from the stellate ganglia using a previously published method [^10^](#_ENREF_10). The stellate ganglia were removed from SD rats at 4 weeks of age and transferred into cold L-15 medium and desheathed carefully under a dissection microscope to remove all surrounding connective tissue. Ganglia were cut into a number of pieces and digested with collagenase type IV (1mgl1ml) and trypsin TRL 3 (2mg/1ml). The ganglia were then rinsed twice in L-15 blocking medium (96.8% L-15 medium supplemented with 0.6% D-(+)-Glucose solution, 2 mmol/L L-glutamine, 100 units/ml penicillin, 100 ug/ml streptomycin, 10% Fetal bovine serum), and rinsed two more times in L-15 plating medium (L-15 incomplete medium, 90% (v/v); NaHCO^3^, 24mM; Glucose, 38mM; Penicillin (10,000 units/ml), 50 units/ml; Streptomycin (10,000 µg/ml), 50 µg/ml; Nerve growth factor, 25ng/ml; Foetal bovine serum, 10% (v/v)) to remove any residual fetal bovine serum. The ganglia were dissociated by sequential mechanical trituration using a fire-polished glass pipettes. Dissociated neurons were plated onto poly-D-lysine/ laminin coated 6 mm cover slips and cultured in L-15 plating medium, then kept at 37.C in 5% CO2. Media were changed every day and experiments were performed 2-3 days after plating.

***Measurement of Norepinephrine Uptake Rate***

NET transporter function was measured using a commercially available assay (neurotransmitter transporter uptake assay; NTUA, Molecular devices: Sunnyvale, CA) by a method previously described [^10^](#_ENREF_10). Prior to the start of the experiment cultured neurons were pre-incubated in a low 1:100 concentration of the assay in L-15 based cell plating medium for 20 min, 37^o^C, 5% CO2. Cells expressing the transporter were selected if a basal level of fluorescence was observed. They were then transferred to a temperature controlled (37^o^C) gravity fed perfusion chamber (volume 500μl; flow rate 3ml/min) on the stage of a Nikon Eclipse TE200-U microscope. Images were acquired every 2 seconds using a photometrics CoolSNAP HQ2 camera, excitation wave length 440nm, emissions band 535±35nm and the rate of increase in intracellular fluorescence recorded. Each experiment had three incubations with NTUA and measurements of NET rate, S1, always control, S2 in the presence or absence of CNP, and S3 always in the presence of the NET inhibitor desipramine (DMI, 100μM). Results are presented as a percentage of S2/S1, to take into account different basal expression of NET between cells and the effect of CNP on NET compared to the time control. Any experiment in which S3 fluorescence increase was not blocked by DMI was excluded from analysis (<5% of cells) as any previous fluorescence increase could not be guaranteed to be NET specific.

***Immunohistochemistry***

Immunohistochemistry of cultured stellate ganglion neurons was performed as previously described [^11^](#_ENREF_11). Anti-tyrosine hydroxylase (TH, mouse) primary antibody (Sigma, 1:200) and biotinylated horse anti-mouse (Santa Cruz, 1:200) secondary antibodies were used on cultured stellate ganglion neurons, which were further labeled with streptavidin Texas red. To assess natriuretic peptide receptor type-B (NPR-B) co-localization, the preparations were blocked using a streptavidin kit, and then incubated with anti-NPR-B (rabbit) primary antibody (Abcam, 1:200) and biotinylated donkey anti-rabbit secondary antibody (Santa Cruz, 1:200) and then labeled with streptavidin fluorescein. Cultured stellate ganglion neurons were also stained with DAPI (1:1000) to illustrate nuclear staining. Tissue was scanned and digitally photographed at x20 magnification using a Nikon Eclipse TE2000-U inversion fluorescence light microscope and appropriate filters.

***Patch-clamp Recordings.***

Whole-cell patch clamp recording was performed at 36±0.5 °C as method previously described [^11^](#_ENREF_11). Pipettes had tip resistances of ~1.5 – 2 MΩ when filled with the internal solution containing (in mM) 140 CsCl, 10 HEPES, 0.1 CaCl2, 4 MgATP, 1 MgCl2 and 1 EGTA, pH adjusted to 7.3 with CsOH. Cultured neurons were superfused with an external solution that designed to isolated whole cell Ca2+ current (containing (in mM) 145 TEACl, 10 HEPES, 4.5 KCl, 1 MgCl2, 11 Glucose, 1 NaHCO3, 2 BaCl2, and 0.001 TTX, pH adjusted to 7.4 with Sigma base 7-9). The bath was grounded by an Ag/AgCl electrode connected via a 3 M KCl/agar bridge. Calcium currents were acquired using Clampex software via an Axopatch 200B amplifier. Series resistance was compensated between 75% and 90%. Current-voltage (I-V) relations were elicited from a holding potential of -90 mV using 50-ms steps (5 s between steps) to test potentials over the range of -50 to +50 mV in 10-mV increment.

***Measurement of Free Intracellular Calcium Concentration****.*

Intracellular Ca^2+^ concentration ([Ca^2+^]I) was determined in single cultured neurons using Fura-2 acetoxymethyl ester (Fura-2/AM, 2μM) by a method previously described [^12^](#_ENREF_12). Loaded neurons were imaged with a QICLICK digital CCD camera (Photometrics) connected to an OptoLED fluorescence imaging system (Cairn Research Ltd) housed on an inverted Nikon microscope equipped with a 40x oil-immersion objective. The cover slip containing the neurons was placed into a temperature-controlled (36±0.5 °C), gravity fed, perfusion chamber (volume: 100 μl), perfused with Tyrode solution at a flow rate of 2 ml/min. [Ca^2+^]I transient was evoked by 30 s exposure to 50 mM KCl (with equimolar reduction in NaCl) in the Tyrode solution. Fura-2AM was excited alternately at 355 nm and 380 nm and the emitted at 510 nm. Fluorescence excitation ratios were transformed into [Ca^2+^]I concentrations using the equation derived by Grynkiewicz et al [^13^](#_ENREF_13). [Ca^2+^]i=Kd×(Sf2/Sb2)×(R−Rmin)/(Rmax−R)

***Reagents and Materials***

Natriuretic peptides were obtained from Calbiochem and P19 from Phoenix Pharmaceuticals. All other reagents were supplied from Sigma-Aldrich if not stated otherwise.

**References**

1. Langenickel TH, Buttgereit J, Pagel-Langenickel I, Lindner M, Monti J, Beuerlein K, Al-Saadi N, Plehm R, Popova E, Tank J, Dietz R, Willenbrock R, Bader M. Cardiac hypertrophy in transgenic rats expressing a dominant-negative mutant of the natriuretic peptide receptor B. *Proc Nat Acad Sci* 2006;**103**:4735-4740.

2. Popova E, Krivokharchenko A, Ganten D, Bader M. Efficiency of transgenic rat production is independent of transgene-construct and overnight embryo culture. *Theriogen* 2004;**61**:1441-1453.

3. Barmashenko G, Buttgereit J, Herring N, Bader M, Ozcelik C, Manahan-Vaughan D, Braunewell KH. Regulation of hippocampal synaptic plasticity thresholds and changes in exploratory and learning behavior in dominant negative NPR-B mutant rats. *Front Mol Neurosci* 2014;**7**:95.

4. Hilzendeger AM, Goncalves AC, Plehm R, Diedrich A, Gross V, Pesquero JB, Bader M. Autonomic dysregulation in ob/ob mice is improved by inhibition of angiotensin-converting enzyme. *J Molecular Med* 2010;**88**:383-390.

5. Gross V, Tank J, Obst M, Plehm R, Blumer KJ, Diedrich A, Jordan J, Luft FC. Autonomic nervous system and blood pressure regulation in RGS2-deficient mice. *Am J Physiol Reg Int Comp Physiol* 2005;**288**:R1134-1142.

6. Bohm M, Lee M, Kreutz R, Kim S, Schinke M, Djavidani B, Wagner J, Kaling M, Wienen W, Bader M, et al. Angiotensin II receptor blockade in TGR(mREN2)27: effects of renin-angiotensin-system gene expression and cardiovascular functions. *J Hyperten* 1995;**13**:891-899.

7. Shanks J, Manou-Stathopoulou S, Lu CJ, Li D, Paterson DJ, Herring N. Cardiac sympathetic dysfunction in the prehypertensive spontaneously hypertensive rat. *Am J Physiol Heart Circ Physiol* 2013;**305**:H980-986.

8. Lee CW, Li D, Channon KM, Paterson DJ. L-arginine supplementation reduces cardiac noradrenergic neurotransmission in spontaneously hypertensive rats. *J Mol Cell Cardiol* 2009;**47**:149-155.

9. Ryan BJ, Lourenco-Venda LL, Crabtree MJ, Hale AB, Channon KM, Wade-Martins R. alpha-Synuclein and mitochondrial bioenergetics regulate tetrahydrobiopterin levels in a human dopaminergic model of Parkinson disease. *Free Rad Biol Med* 2014;**67**:58-68.

10. Shanks J, Mane S, Ryan R, Paterson DJ. Ganglion-specific impairment of the norepinephrine transporter in the hypertensive rat. *Hypertension* 2013;**61**:187-193.

11. Li D, Lu CJ, Hao G, Wright H, Woodward L, Liu K, Vergari E, Surdo NC, Herring N, Zaccolo M, Paterson DJ. Efficacy of B-Type Natriuretic Peptide Is Coupled to Phosphodiesterase 2A in Cardiac Sympathetic Neurons. *Hypertension* 2015;**66**:190-198.

12. Li D, Lee CW, Buckler K, Parekh A, Herring N, Paterson DJ. Abnormal intracellular calcium homeostasis in sympathetic neurons from young prehypertensive rats. *Hypertension* 2012;**59**:642-649.

13. Grynkiewicz G, Poenie M, Tsien RY. A New Generation of Ca-2+ Indicators with Greatly Improved Fluorescence Properties. *J Biol Chem* 1985;**260**:3440-3450.
